# MMP-10 from M1 macrophages promotes pulmonary vascular remodeling and pulmonary arterial hypertension

**DOI:** 10.7150/ijbs.66472

**Published:** 2022-01-01

**Authors:** Pei-Ling Chi, Chin-Chang Cheng, Cheng-Chung Hung, Mei-Tzu Wang, Hsien-Yueh Liu, Meng-Wei Ke, Min-Ci Shen, Kun-Chang Lin, Shu-Hung Kuo, Pin-Pen Hsieh, Shue-Ren Wann, Wei-Chun Huang

**Affiliations:** 1 Department of Medical Education and Research, Kaohsiung Veterans General Hospital, Kaohsiung City 81362, Taiwan; 2 Department of Pathology and Laboratory Medicine, Kaohsiung Veterans General Hospital, Kaohsiung, Taiwan; 3 Department of Critical Care Medicine, Kaohsiung Veterans General Hospital, Kaohsiung 81362, Taiwan; 4 Bachelor Degree Program in Animal Healthcare, Hungkuang University, Taichung City, Taiwan; 5 The Agricultural College, Tunghai University, Taichung City, Taiwan; 6 Department of Anatomic Pathology, Dalin Tzu Chi Hospital, Buddhist Tzu Chi Medical Foundation, Chia-yi, Taiwan; 7 School of Medicine, Tzu Chi University, Hualien, Taiwan; 8 Pingtung Branch, Kaohsiung Veterans General Hospital, Pingtung County, Taiwan; 9 Department of Physical Therapy, Fooyin University, Kaohsiung, Taiwan; 10 School of Medicine, National Yang-Ming University, Taipei, Taiwan; 11 Graduate Institute of Clinical Medicine, Kaohsiung Medical University, Kaohsiung, Taiwan

**Keywords:** MMP-10, pulmonary arterial hypertension, M1 macrophges, vascular remodeling, pulmonary arterial smooth muscle cells

## Abstract

Pulmonary arterial hypertension (PAH) is characterized by muscularized pulmonary blood vessels, leading to right heart hypertrophy and cardiac failure. However, state-of-the-art therapeutics fail to target the ongoing remodeling process. Here, this study shows that matrix metalloproteinases (MMP)-1 and MMP-10 levels are increased in the medial layer of vessel wall, serum, and M1-polarized macrophages from patients with PAH and the lungs of monocrotaline- and hypoxia-induced PAH rodent models. MMP-10 regulates the malignant phenotype of pulmonary artery smooth muscle cells (PASMCs). The overexpression of active MMP-10 promotes PASMC proliferation and migration via upregulation of cyclin D1 and proliferating cell nuclear antigen, suggesting that MMP-10 produced by infiltrating macrophages contributes to vascular remodeling. Furthermore, inhibition of STAT1 inhibits hypoxia-induced MMP-10 but not MMP-1 expression in M1-polarized macrophages from patients with PAH. In conclusion, circulating MMP-10 could be used as a potential targeted therapy for PAH.

## Introduction

Pulmonary arterial hypertension (PAH) is an extraordinarily complex and multifaceted vascular disease caused by several risk factors that lead to a variety of pathological insults. Histopathological examination of PAH shows progressive narrowing of the distal pulmonary arteries and muscularized vessels, resulting from the uncontrolled proliferation of pulmonary artery smooth muscle cells (PASMCs), pulmonary artery endothelial cells (PAECs), and fibroblasts, which leads to right heart hypertrophy and eventually cardiac failure 1, 2. This suggests that an imbalance in the cross-talk between PASMCs, PAECs, and fibroblasts plays a fundamental role in vascular remodeling and is involved in the development and progression of PAH.

Vascular remodeling occurs early in the PAH disease process and involves not only vascular smooth muscle cell (VSMC) proliferation but also excessive extracellular matrix (ECM) modulation, which is a component of the thickened pulmonary vascular media 3. ECM components degraded by matrix metalloproteinases (MMPs) such as MMP-1, MMP-2, MMP-3, and MMP-9, which can be produced by SMCs and macrophages, promote the proliferation and migration of VSMCs, resulting in increased vascular stiffness or reduced pulmonary arterial compliance 2, 4. Elevated levels of MMP-2 and tissue inhibitor matrix metalloproteinase-1 (TIMP-1), but decreased levels of MMP-3 were found in human lung tissues from patients with PAH 5. Zervoudaki et al. identified higher plasma levels of MMP-2 and MMP-9 in patients with PAH 6. In experimental PAH models, microarray analysis demonstrated the upregulation of MMP-2, 8, 9, 10, 11, 12, and 20 involved in ECM regulation in monocrotaline (MCT)-induced rats 7.

Although dysregulation of MMPs may be involved in vessel remodeling and the development of PAH, there is still a lack of information about the expression profile of MMPs in patients with PAH. Furthermore, most studies have focused on the expression of MMPs at the mRNA level in lung tissues or analyzed the serum of patients with PAH. Few studies have clarified the specific distribution of MMPs in medial VSMCs, adventitial microvessels, or infiltrated macrophages.

Macrophages have also been reported to be potential sources of MMPs, a secreted enzyme that can digest the vascular matrix, resulting in vascular remodeling 2. Based on the patterns of gene and protein expression, activated macrophages are routinely classified as M1 or M2 macrophages. M1 macrophages induced by infection with an acute pro-inflammatory phenotype and polarization *in vitro* are driven by low concentrations of lipopolysaccharide (LPS) and IFN-γ. However, M2 macrophages activated by IL-4 or IL-10 are anti-inflammatory and immunoregulatory 8. There was no difference in MMP-1 expression in PASMCs between controls and PAH patients 9; however, cells co-cultured with monocyte-derived macrophages exhibited an increase in MMP-1 expression, suggesting that infiltrated macrophages may play a role in the regulation of MMPs and that the additional effects of MMPs on PASMCs may be important in the early stages of vascular remodeling.

In the present study, we analyzed the presence of MMPs in patients with PAH as well as investigated their expression in different macrophage subtypes. We also assessed the role of MMPs in the biological behavior of PASMCs. Our study presents MMP-10 as an indicator of signal transducer and activator of transcription 1 (STAT1) in STAT1-targeted therapy for PAH.

## Results

### Elevated pulmonary and circulating levels of MMP-1 and MMP-10 in patients with PAH

MMPs are known to regulate the proliferation, migration, and apoptosis of vascular ECs and SMCs, which are involved in the progression of PAH 5. Perivascular inflammatory cell infiltrates are seen in plexiform lesions in the lungs of patients with PAH 10. To further explore whether MMP expression levels correlate with macrophage infiltration in lung tissue, we determined the cell types contributing to the production of MMPs and analyzed the expression profile of 10 MMPs in PAH patients. Freshly isolated monocytes from patients with PAH or healthy donors was used to differentiated into M0, M1, or M2 phenotypes. PAH patients (n=39) and healthy individuals as controls (n=30) were included in the study. The demographic and clinical data, hemodynamic measures, exercise capacity parameters, and baseline characteristics of the two groups are shown in **Table 1**. The mean age of the PAH and the control groups was 54.0 ± 18.2 (mean ± SD) and 56.3 ± 13.7 years, respectively. No significant differences were observed in any of the baseline characteristics.

**Figure 1:**
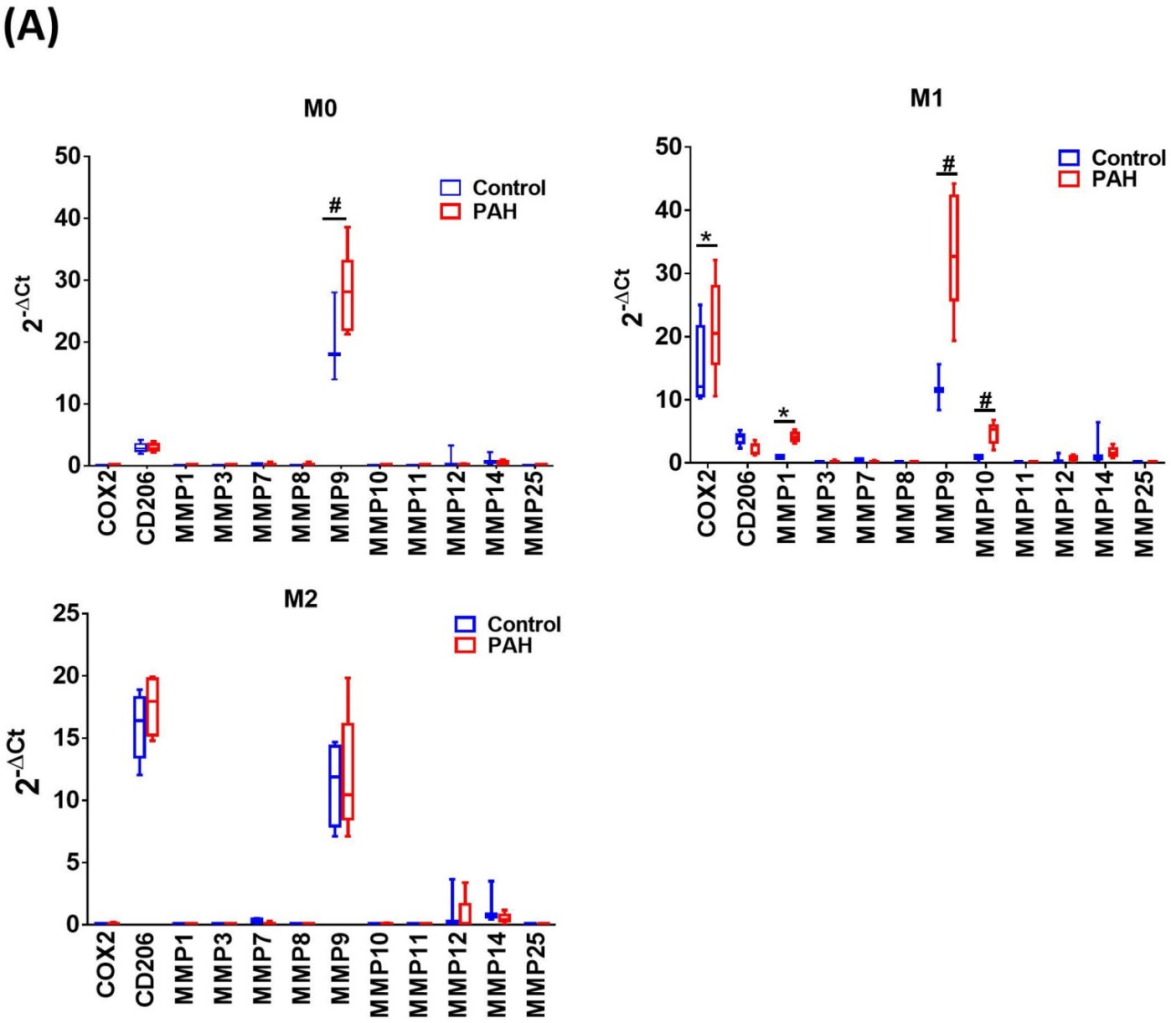

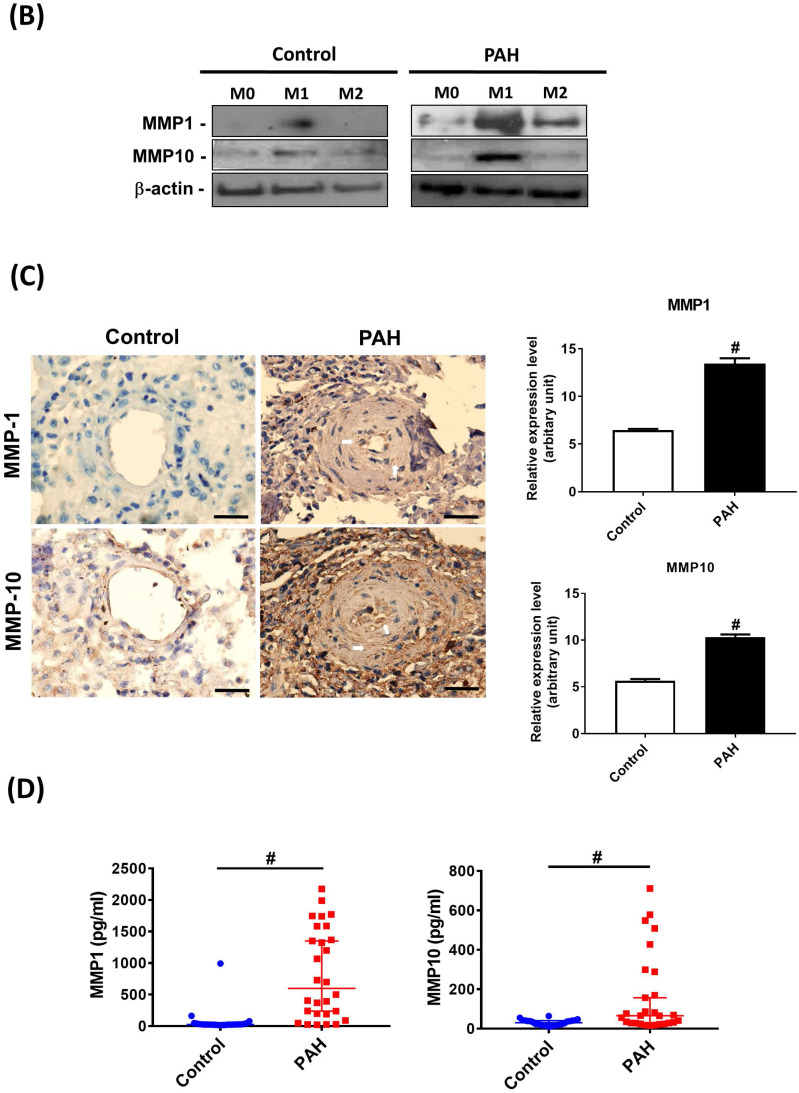
** Increased expression levels of MMP-1 and MMP-10 in PAH patients.** (A) Basal gene expression levels of MMPs were measured in M0, M1- and M2- differentiated macrophages derived from blood of patients with PAH (n=39) and healthy controls (n=30). Values were normalized to GAPDH and expressed as 2^-∆CT^ values±SD. *P < 0.05 and^ #^*P <* 0.01 compared with healthy control. (B) M0, M1- and M2- differentiated macrophages derived from blood of patients with PAH and healthy controls were lysed and subjected to western blotting to measure the protein levels of MMP-1 and MMP-10. (C) Immunohistochemistry of MMP-1 an dMMP-10 in lung tissue sections of patients with PAH and the control donors. Brown color indicates the staining of MMP-1 and MMP-10. White arrows indicate the elevated of MMP-1 and MMP-10 staining in the PASMC layer of the vessels. Scale bars, 100 μm. Representative the quantifications (right) of MMP-1 and MMP-10 protein in lung tissue sections of patients with PAH and the control donors. (D) Serum levels of MMP-1 and MMP-10 were assessed by ELISA. Group comparisons were analyzed by one-way analysis of variance (ANOVA) with Dunnett's post-hoc test. Data are represented as the mean ±SD. ^*^P < 0.05 and ^#^*P* < 0.01, PAH patient vs healthy control.

Upon M-CSF-mediated differentiation into macrophages (M0), the expression of MMP-9 mRNA in the PAH group was significantly higher than that in the control group. No significant differences in the expression of other MMP mRNAs were observed between the two groups. In the M1- and M2-polarized macrophages, there was no difference in the expression of the M2 (CD206) marker between the PAH and control groups. However, the M1 (COX-2) marker was more highly expressed in M1-polarized macrophages from the PAH group than that in the control group. Moreover, we found that MMP-1, MMP-9, and MMP-10 mRNA levels in M1-polarized macrophages from PAH were significantly higher than those in controls, while the other MMPs did not show any significant differences between the two groups. In M2-polarized macrophages, we observed no difference in MMP expression between the PAH and control groups (**Fig. 1A**). Similarly, M1-polarized macrophages from patients with PAH, but not from healthy controls, expressed markedly increased protein levels of MMP-1 and MMP-10 compared to M0- and M2-polarized macrophages (**Fig. 1B**). Immunohistochemical imaging of lung specimens from PAH patients revealed significantly enhanced immunoreactivity for MMP-1 and MMP-10 in lung sections as compared to that in the control group (**Fig. 1C**). In addition to MMP-1 and MMP-10 expression in the lung tissue, we assessed the circulating levels of MMP-1 and MMP-10 in PAH patients. Serum MMP-1 and MMP-10 levels were markedly increased in patients with PAH compared to those in healthy individuals (**Fig. 1D**). These results suggest the upregulation of MMP-1 and MMP-10 in PAH.

### Increased expression of MMP-1 and MMP-10 in the infiltrating macrophages from the lungs of MCT-induced rats

**Figure 2:**
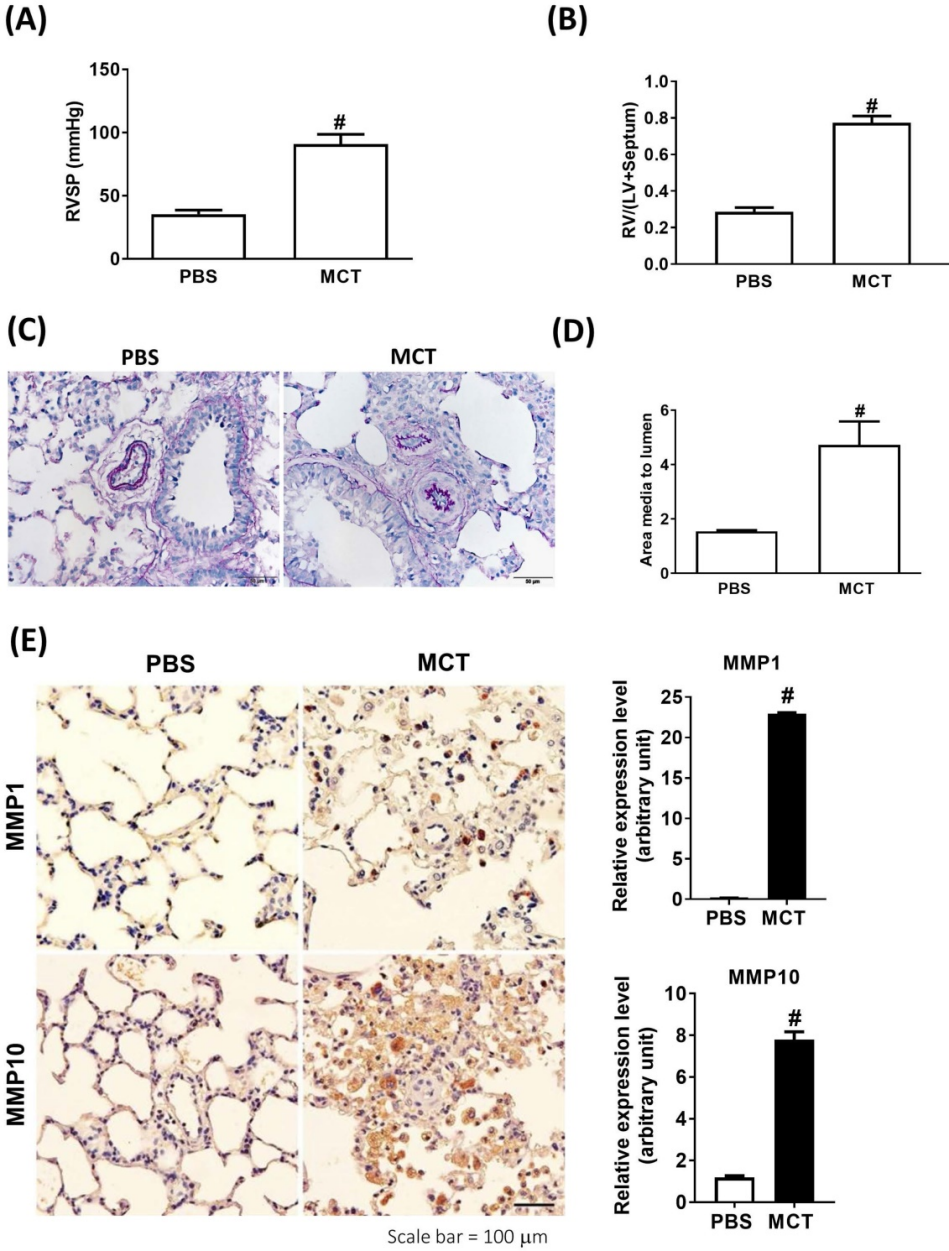

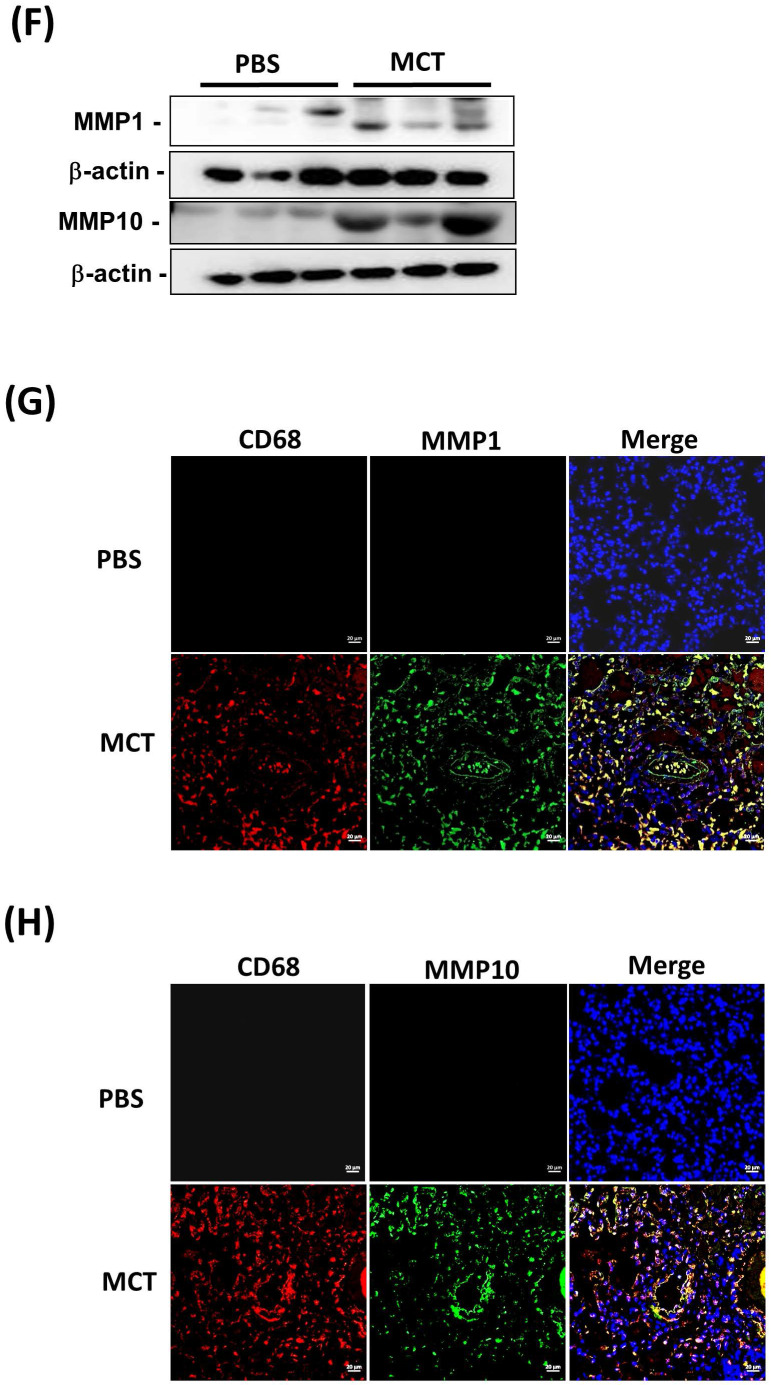
** Increased expression levels of MMP-1 and MMP-10 in MCT-induced PAH rat lungs.** Rats were treated with MCT (60 mg/kg) or PSB for 28 days (n=10 per group). (A) Assessment of right ventricular systolic pressure (RVSP), (B) right ventricular hypertrophy (Fulton index, the ratio of right ventricular weight to left ventricular plus septal weight. (C) Lung sections were stained for Elastic van Gieson (EvG). Scale bars, 50 μm. (D) The ratio of media thickness to lumen diameter were determined using Image J analysis software. Group comparisons were analyzed by one-way analysis of variance (ANOVA) with Dunnett's post-hoc test. Data are represented as the mean ±SD. ^#^*P* < 0.01, MCT vs PBS group. (E) Immunohistochemistry of MMP-1 and MMP-10 in lung tissue sections of MCT-rats and the control. Scale bars, 100 μm. Representative the quantifications (right) of MMP-1 and MMP-10 protein in lung tissue sections of MCT-rats and the control. (F) Whole lung tissues were isolated from MCT-induced PAH rats and western blotting was used to measure the level of MMP-1 and MMP-10. (G, H) Immunofluorescence for the macrophage markers CD68 (red) and MMP-1 (green) or MMP-10 (green) and nuclei with DAPI dye (blue) in lung sections. Scale bars, 20 µm.

We further analyzed the expression of MMP-1 and MMP-10 in the lung tissue samples obtained from an experimental PAH model of PAH established by challenging rats with MCT. Administration of MCT resulted in elevated right ventricular systolic pressure (RVSP) and right ventricular hypertrophy, as indicated by the significantly increased right ventricle (RV)/[left ventricle (LV) + septum (S)] ratio (**Fig. 2A and B**), confirming that MCT could effectively induce PAH-like symptoms.

Similar to the findings in human PAH lung tissues, EVG staining showed an increase in the muscularized and occluded pulmonary arteries in MCT-induced rats compared with those of the control group. The immunoreactivity of MMP-1 and MMP-10 was significantly elevated in the lungs of MCT-induced hypertensive rats (**Fig. 2C**). The media thickness and the ratio of the media thickness to lumen diameter were significantly increased in the distal pulmonary arteries of MCT-treated rats (**Fig. 2D**), further confirming the development of PAH. Immunohistochemistry also showed significantly increased expression of MMP-1 and MMP-10 in the lungs of MCT-induced hypertensive rats (**Fig. 2E**). Consistently, western blotting showed that MMP-1 and MMP-10 protein levels were increased in the whole lung tissues isolated from MCT-induced rats compared with those of controls (**Fig. 2F**). Double labeling with CD68 (a macrophage marker) and either MMP-1 or MMP-10 revealed that macrophages were the major source of MMP-1 and MMP-10 in the lungs of MCT-induced rats (**Fig. 2G and H**). No signals for CD68, MMP-1, and MMP-10 were detected in the lung sections of control rats, suggesting an increased influx of macrophages in MCT-induced rats. This finding further supported that these recruited macrophages express MMP-1 and MMP-10.

### Increased expression of MMP-1 and MMP-10 in the infiltrating macrophages from the lungs of hypoxia-induced rats

**Figure 3:**
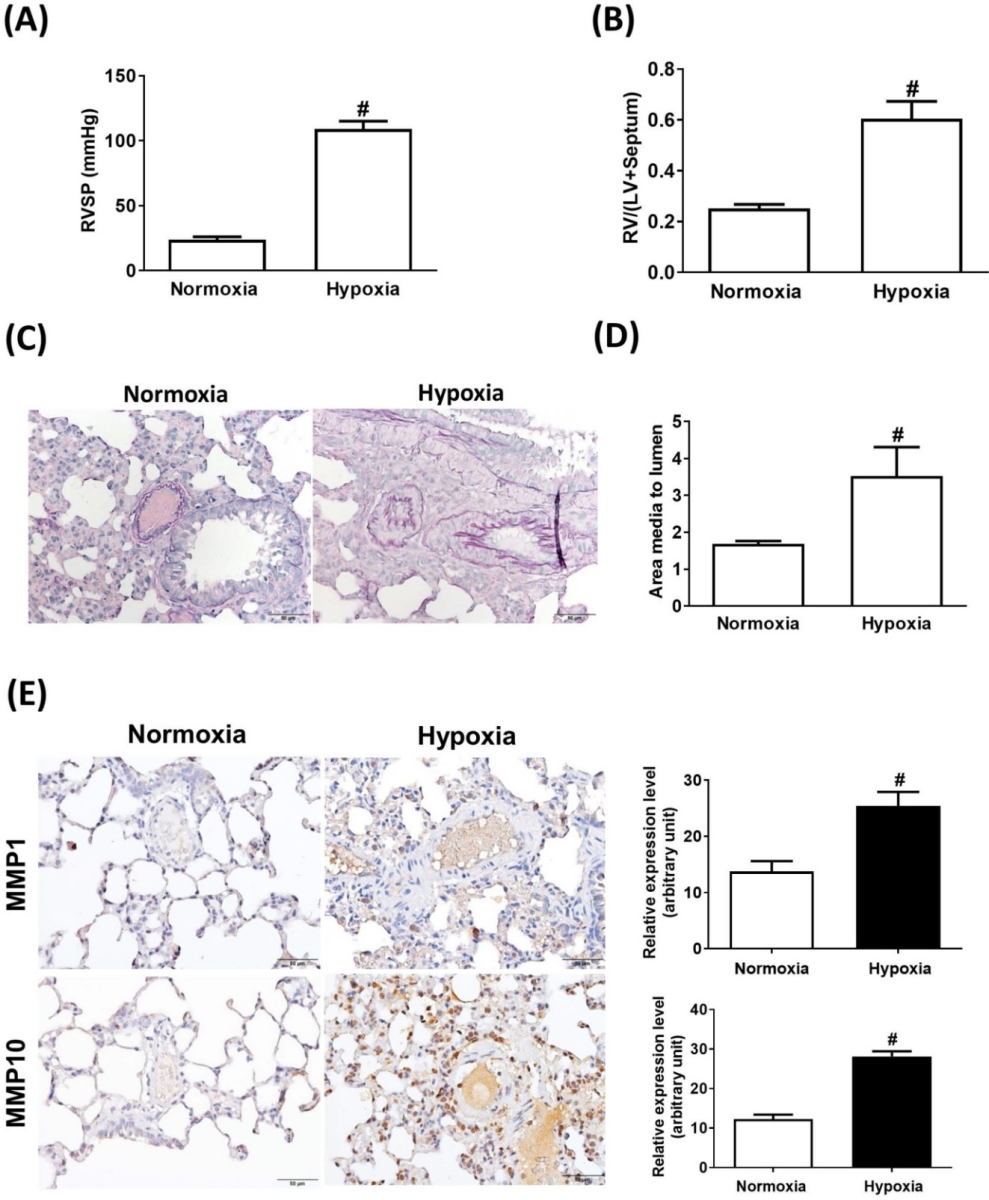

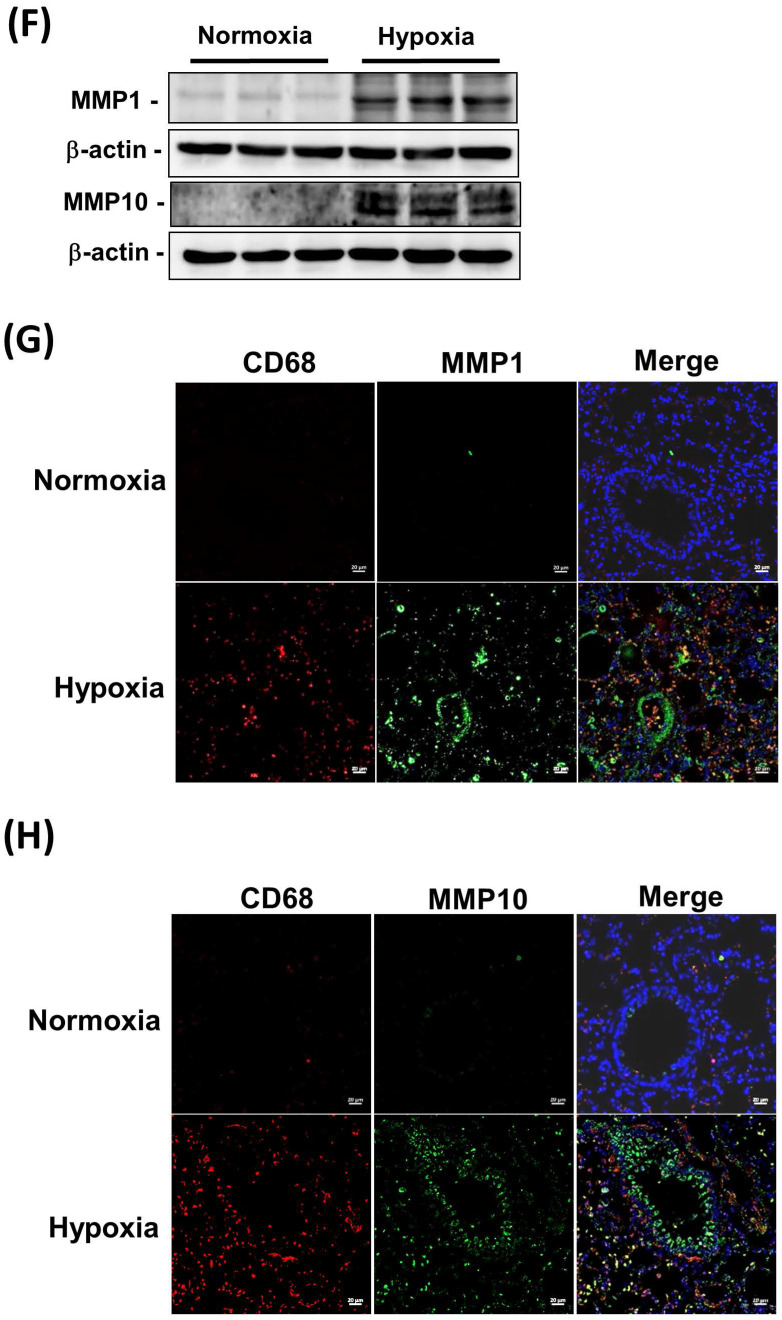
** Increased expression levels of MMP-1 and MMP-10 in hypoxia-induced PAH rat lungs.** Rats were exposed to normoxia or hypoxia (10% O_2_) for 8 weeks (n=10 per group). (A) RVSP, (B) right ventricular hypertrophy were analyzed. (C) Lung sections were stained for EvG. Scale bars, 50 μm. (D) The ratio of media thickness to lumen diameter were determined using Image J analysis software. Group comparisons were analyzed by one-way analysis of variance (ANOVA) with Dunnett's post-hoc test. Data are represented as the mean ±SD. ^#^*P* < 0.01, hypoxia vs normoxia group. (E) Immunohistochemistry of MMP-1 and MMP-10 in lung tissue sections of hypoxic rats and the control. Scale bars, 100 μm. Representative the quantifications (right) of MMP-1 and MMP-10 protein in lung tissue sections of hypoxic rats and the control. (F) Whole lung tissues were isolated from hypoxia-induced PAH rats and western blotting was used to measure the level of MMP-1 and MMP-10. (G, H) The lung sections of rats from each group were co-immunostained with CD68 and MMP-1 or MMP-10 antibodies and DAPI. Scale bars, 20 µm.

Sustained exposure to hypoxia leads to hypoxic vasoconstriction, resulting in the development of increased pulmonary vascular resistance and pulmonary hypertension 11; thus, structural changes in the pulmonary vasculature are the major determinants of elevated vascular resistance. To investigate whether the hypoxic condition was responsible for the increase in MMP-1 and MMP-10 expression in the lungs, the rats were maintained under a hypoxic condition for 8 weeks. Hypoxia induced increases in the RVSP (**Fig. 3A**), RV/(LV + S) ratio (**Fig. 3B**), and pulmonary arterial wall thickness (**Fig. 3C** and **D**) compared with those in the normoxic group. Similar to the findings in human PAH lungs and the MCT-induced PAH rat model, the protein levels of MMP-1 and MMP-10 were significantly elevated in hypoxia-treated rat lungs as measured by immunohistochemistry and western blotting (**Fig. 3E** and **F**). Double immunostaining with anti-CD68 and anti-MMP-1 or anti-MMP-10 antibodies confirmed that MMP-1 and MMP-10 were expressed in the macrophages infiltrating the lung tissues of hypoxia-treated rats.

### Overexpression of active MMP-10 promotes proliferative and pro-migratory phenotypes of human PASMCs

**Figure 4:**
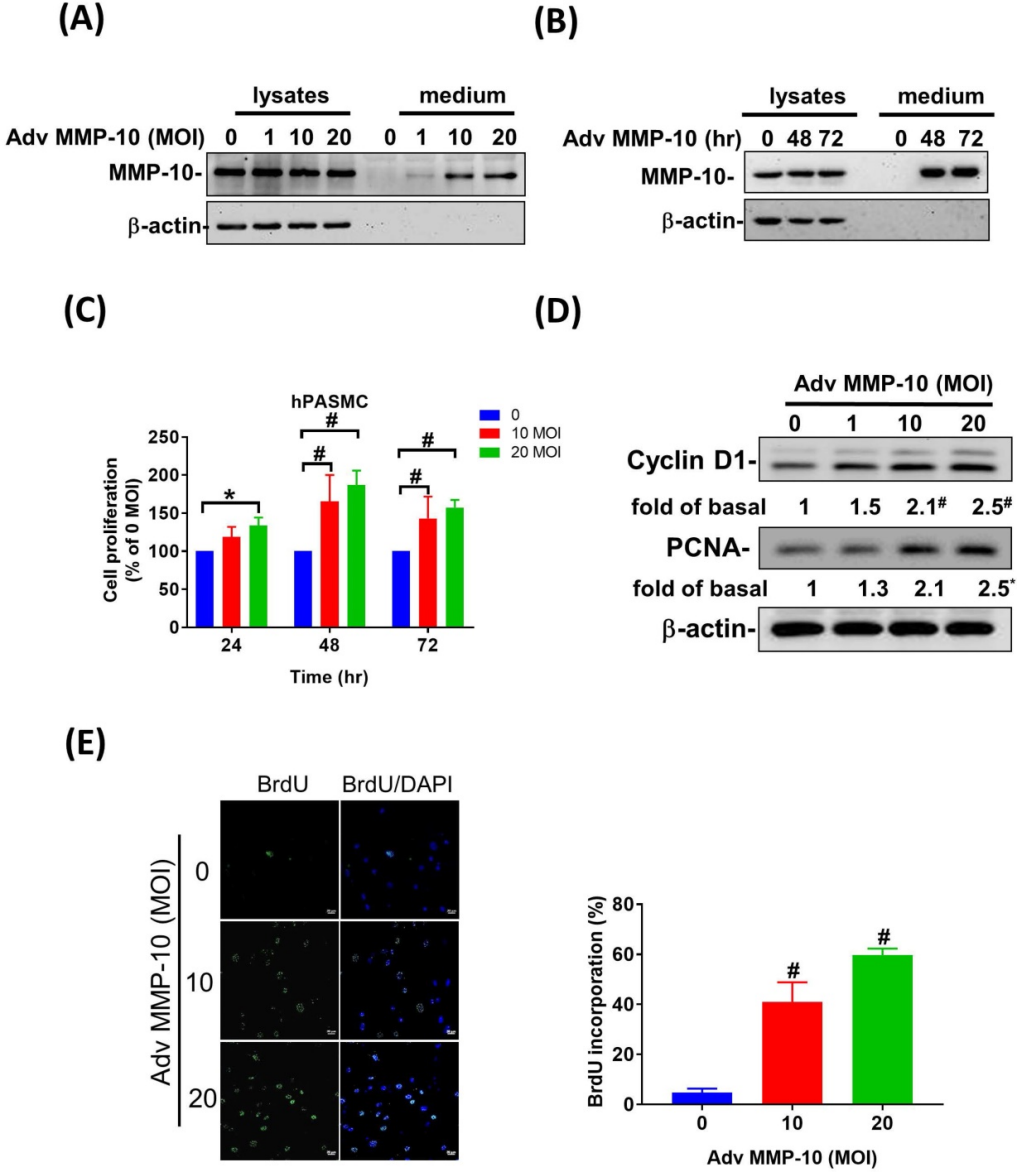

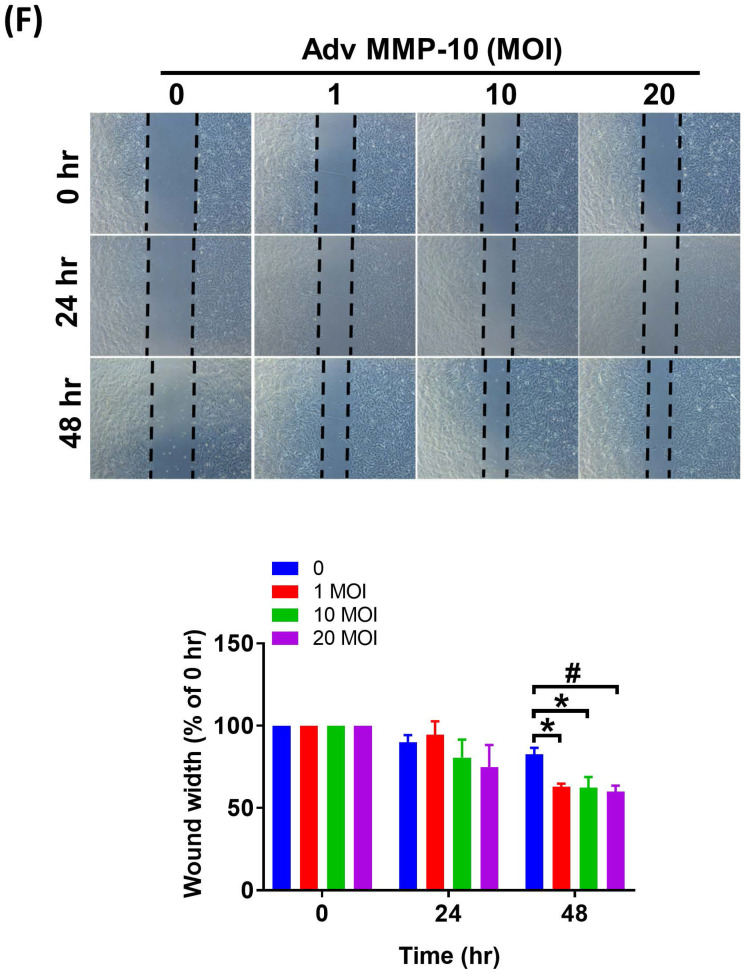
** MMP-10 promotes proliferation and migration in human PASMC.** (A) Human PASMC were infected with 0, 1, 10, or 20 MOI of Adv-MMP-10, an adenovirus containing human MMP-10 gene, for 48 h. (B) Cells were infected with 10 MOI of Adv-MMP-10 for 48 or 72 h. The expression level of MMP-10 by western blotting in cell lysates and culture medium were determined. (C) Human PASMC were infected with 0, 10 or 20 MOI of Adv-MMP-10 for 24, 48 or 72 h. Cell viability were measured by alamar blue assay. (D) Human PASMC were infected with 0, 1, 10 or 20 MOI of Adv-MMP-10 for 48 h. The expression level of cyclin D1 and PCNA by western blotting in cell lysates were determined. (E) Cell proliferation were measured using BrdU assay. (F) Scratch-wound closure monitored over time in Adv-MMP-10 infected human PASMC. Representative bright-field images and quantification of the wound closure expressed as the remaining area uncovered by the cells. Data are expressed as the mean ± SEM of three independent experiments. ^*^P < 0.05; ^#^*P* < 0.01, as compared with the basal level.

Both PASMC proliferation and migration play vital roles in the progression of pulmonary vascular remodeling in PAH 12. It has been demonstrated that MMP-1 has deleterious effects on the development of pulmonary hypertension via excessive bioavailability of VEGF in the aortic SMCs co-cultured with macrophages, which is associated with pathological lung vascular remodeling in PAH 13, 14. However, little is known about the involvement of MMP-10 in vascular remodeling in PAH. Therefore, to determine the effects of active MMP-10 on the biological behavior of PASMCs, adenovirus-mediated overexpression of active MMP-10 was used. Active MMP-10 was produced in human PASMCs infected with Adv-MMP-10 (10, 20 MOI) for 48 h or infected with Adv-MMP-10 (10 MOI) for 48 and 72 h, as determined by immunoblotting of conditioned medium from serum-starved cells (**Fig. 4A and B**), indicating that the active MMP-10 was significantly upregulated by Adv-MMP-10 infection in human PASMCs. In order to clarify the biological significance of active MMP-10 expression in PASMCs, cell proliferation and migration were studied in relation to active MMP-10 expression. As shown in **Fig. 4C**, there was a significant increase in cell proliferation after infection with Adv-MMP-10 (10, 20 MOI) compared to that in non-infected cells at 48 and 72 h. Next, we examined whether MMP-10 promoted PASMC growth by upregulating the proliferation marker proliferating cell nuclear antigen (PCNA) or cell cycle regulatory protein cyclin D1 (cyclin D1). The western blot analysis showed that overexpression of MMP-10 enhanced the expression of both cyclin D1 and PCNA in human PASMCs (**Fig. 4D**). The role of active MMP-10 in cell proliferation was further confirmed by BrdU incorporation. Similarly, more BrdU-positive cells were detected in PASMCs infected with Adv-MMP-10 than that in non-infected cells (**Fig. 4E**). To determine the functional role of active MMP-10 in regulating PASMC migration, cells were infected with Adv-MMP-10 and a wound scratch healing assay was performed to monitor the wound healing capacity at different time points. As shown in **Fig. 4F**, the distance of the scratching gap was significantly smaller in PASMCs overexpressing active MMP-10 than that in non-infected cells. These results indicate that PASMCs overexpressing active MMP-10 exhibit an enhanced migration and proliferation ability via the increased expression of cyclin D1 and PCNA.

### Overexpression of active MMP-10 promotes the proliferative and pro-migratory phenotypes of PASMCs derived from MCT- and hypoxia-exposed rats

**Figure 5:**
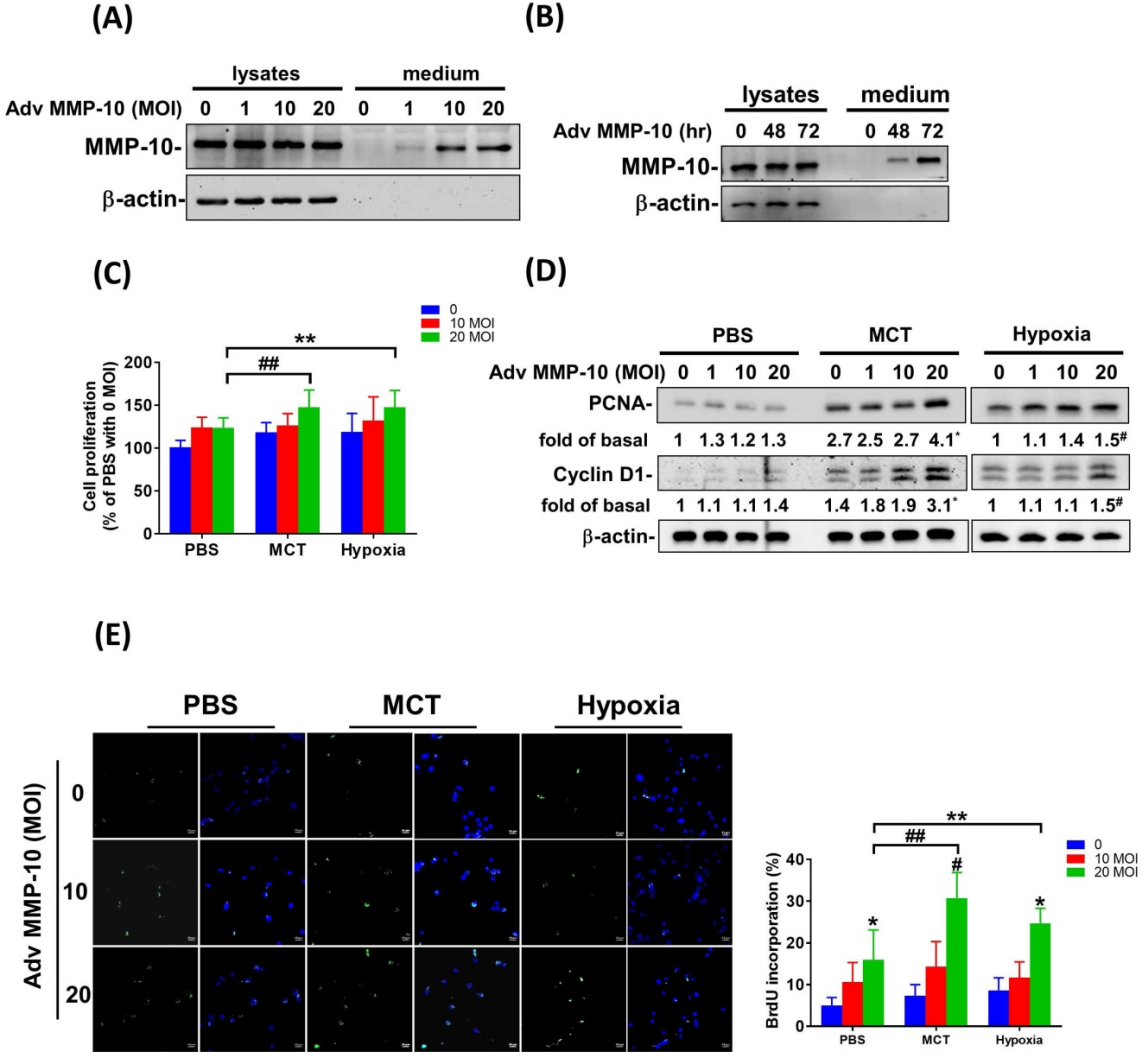

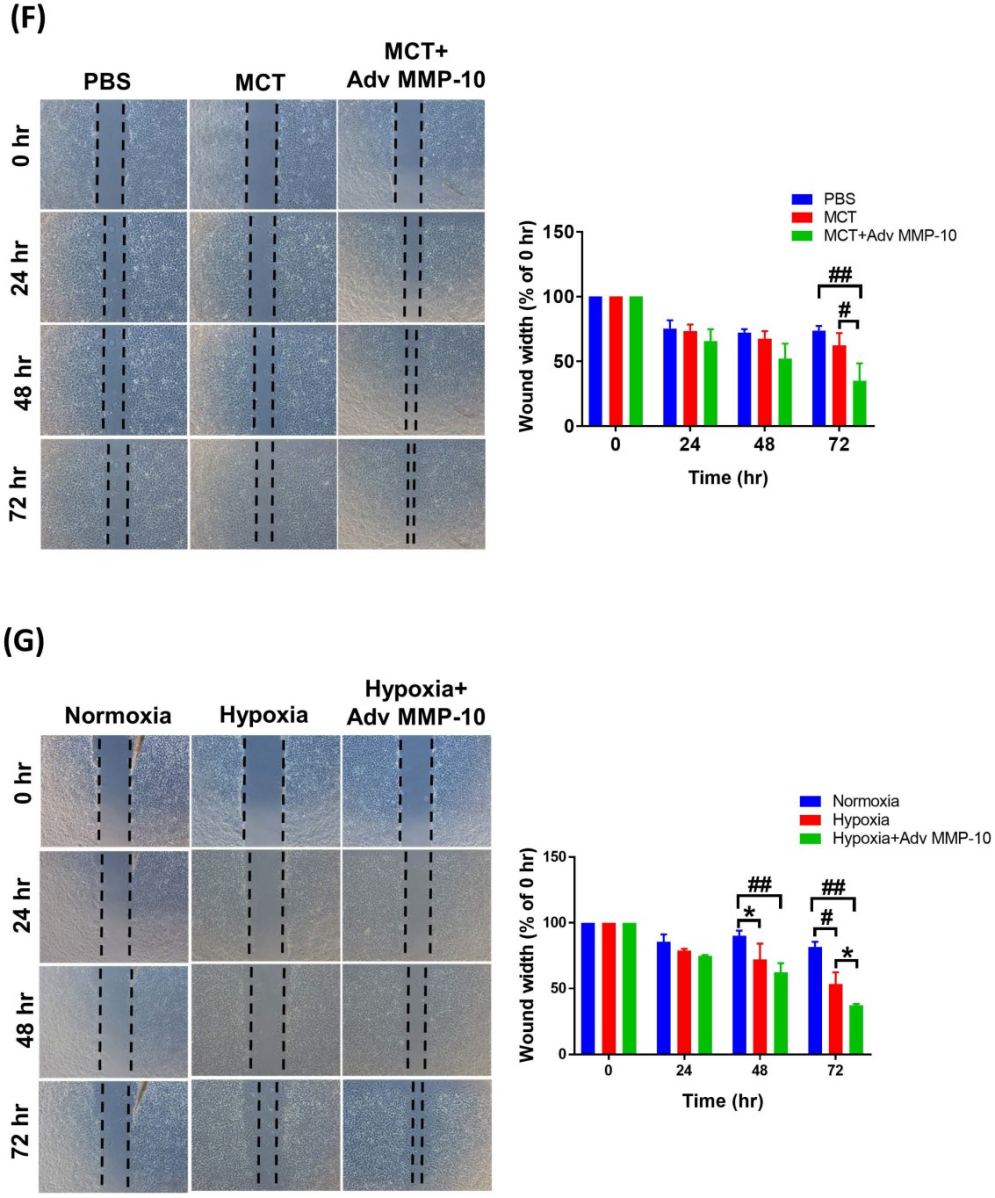
** MMP-10 exacerbates proliferation and migration of rat PAH PASMC.** (A) PASMC isolated from MCT- and hypoxia-induced rats. Cells were infected with 0, 1, 10, or 20 MOI of Adv-MMP-10 for 48 h. (B) Cells were infected with 10 MOI of Adv-MMP-10 for 48 or 72 h. The expression level of MMP-10 by western blotting in cell lysates and culture medium were determined. (C)MCT- and hypoxia-PASMC were infected with 0, 10 or 20 MOI of Adv-MMP-10 for 72 h. Cell viability were measured by alamar blue assay. (D) MCT- and hypoxia-PASMC were infected with 0, 1, 10 or 20 MOI of Adv-MMP-10 for 72 h. The expression level of cyclin D1 and PCNA by western blotting in cell lysates were determined. (E) Cell proliferation were measured using BrdU assay. (F) Scratch-wound closure monitored over time in Adv-MMP-10 infected MCT- and hypoxia-PASMC. Representative bright-field images and quantification of the wound closure expressed as the remaining area uncovered by the cells. Data are expressed as the mean ± SD of three independent experiments. ^*^P < 0.05; ^#^*P* < 0.01, as compared with the basal level. In C, E, F, G, ^**^P < 0.05; ^##^*P* < 0.01, MCT-PASMC/20 MOI Adv-MMP-10 or hypoxia-PASMC/20 MOI Adv-MMP-10 vs PBS-PAMC/20 MOI Adv-MMP-10.

To confirm the role of active MMP-10 in the promotion of the proliferative and pro-migratory phenotypes of PASMCs, rat PASMCs were infected with Adv-MMP-10 (1, 10, 20 MOI) for 48 h. As shown in **Fig. 5A**, active MMP-10 was produced in rat PASMCs with human Adv-MMP-10 (10 and 20 MOI). After infection with Adv-MMP-10 (10 MOI) for 48 and 72 h, PASMCs generated active MMP-10 (**Fig. 5B**). To explore the magnitude of cell behavioral changes, we compared PASMCs from MCT- and hypoxia-treated rats with PASMCs from control rats. We found that the overexpression of active MMP-10 resulted in a significant increase in cell viability of PASMCs derived from MCT- and hypoxia-treated rats compared to that of PASMCs from the control group (**Fig. 5C**). Compared to PASMCs from the control, overexpression of active MMP-10 in the PASMCs from the MCT- and hypoxia-treated group resulted in an enhanced expression of cyclin D1 and PCNA (**Fig. 5D**). BrdU staining further confirmed that BrdU-positive cells were increased following active MMP-10 overexpression in control PASMCs, which is consistent with the findings in human PASMCs. Moreover, overexpression of active MMP-10 in PASMCs from the MCT- and hypoxia-treated group led to a significant increase in the number of BrdU-positive cells compared to that in control PASMCs (**Fig. 5E**). Using the wound healing assay, we observed a significant increase in migration in Adv-MMP-10 infected PASMCs derived from the MCT- and hypoxia treated groups compared to that in PASMCs from the control group (**Fig. 5F and G**). Altogether, these results suggest that the overexpression of active MMP-10 results in a markedly enhanced proliferative and migratory phenotype of rat PAH PASMCs compared to that in the controls.

### Regulation of MMP-1 and MMP-10 expression in M1-polarized macrophages from PAH patients

**Figure 6:**
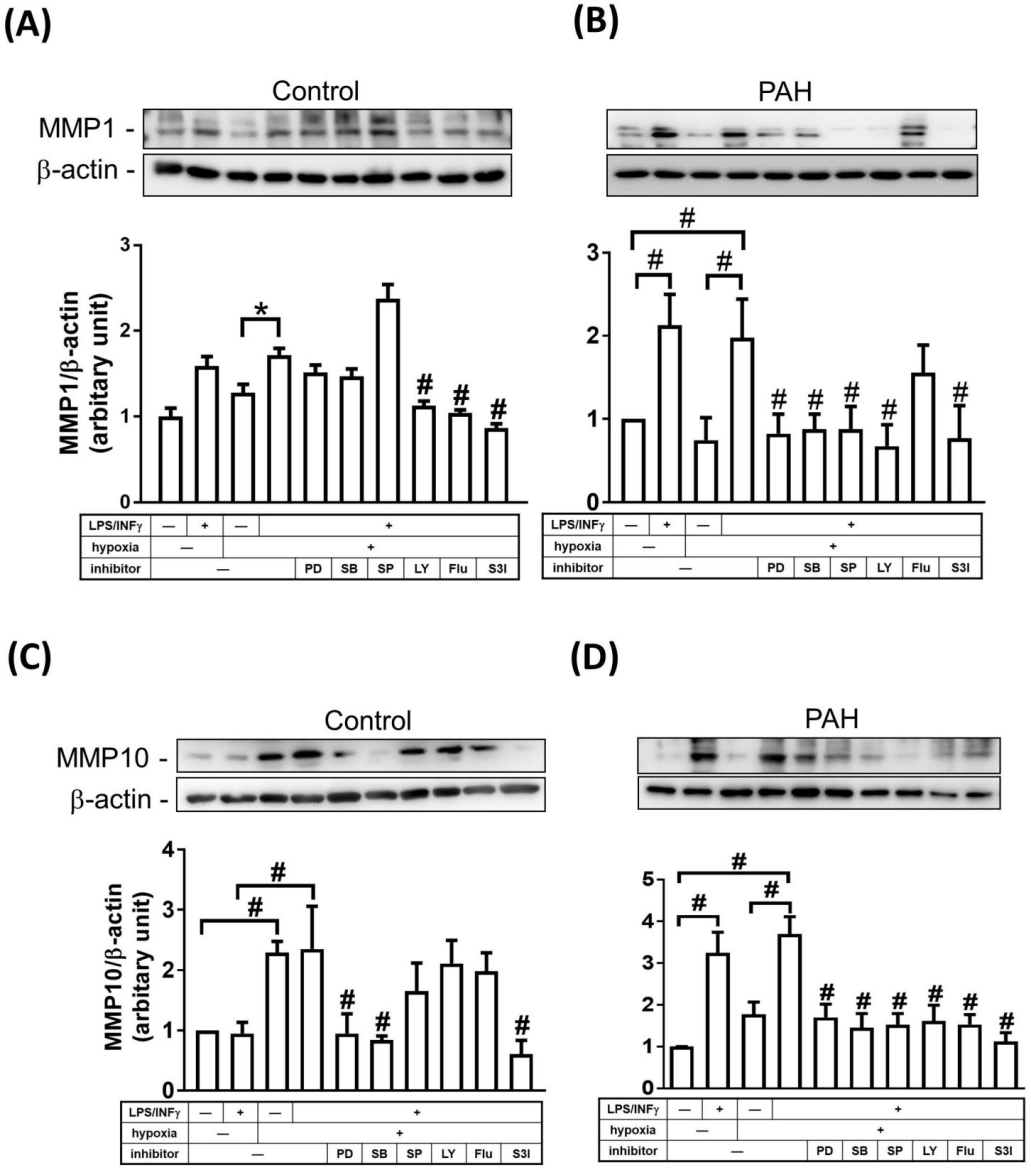
** MAPKs, PI3K, and STATs pathways are involved in the regulation of MMP-1 and MMP-10 expression in M1-polarized macrophages from PAH patients.** (A-D) M0 macrophages cultured in medium without M-CSF for 16 h. Subsequently, cells were pretreated in the absence of inhibitor or with PD98059 (10 μM), SB203580 (10 μM), SP600125 (10 μM), LY-294002 (10 μM), Fludarabine (Flu, 1 μM ,STAT1 inhibitor), or S3I-201 (50 μM, STAT3 inhibitor) for 1h and then incubated in the presence or absence of LPS/INF-γ for 18 h. After stimulation, cells were further exposed either to hypoxia or normoxia for 24 h. The expression level of MMP-10 by western blotting was determined. Data are expressed as the mean ± SD of three independent experiments. ^*^P < 0.05; ^#^*P* < 0.01, as compared with LPS/INF-γ group under hypoxic conditions.

STAT and mitogen-activated protein kinase (MAPK) signaling pathways have been shown to be involved in regulating the expression of MMPs 15, 16. To examine whether MAPKs and STAT regulate MMP-1 and MMP-10 expression in M1 macrophages, cells were treated with inhibitors of MEK-1/2 (U0126), p38 MAPK (SB202190), JNK-1/2 (SP600125), PI3K/AKT (LY294002), STAT1 (Fludarabine), and STAT3 (S3I-201) inhibitors. As shown in **Fig. 6A-D**, M1 macrophages from PAH patients, but not from healthy controls, expressed markedly increased protein levels of MMP-1 and MMP-10 compared to M0- polarized macrophages. To explore the magnitude of MMP changes, we compared normoxic M1-polarized macrophages with hypoxic M1-polarized macrophages. In healthy controls, MMP-1 expression was higher in hypoxic M1 macrophages than that in hypoxic M0 macrophages, whereas the expression of MMP1 in hypoxic M1 macrophages was significantly suppressed by LY294002, STAT1, and STAT3 inhibitors (**Fig. 6A**). Moreover, hypoxia induced MMP-10 expression in M0 and M1 macrophages, while the expression of MMP10 in hypoxic M1 macrophages was significantly suppressed by PD98059, SB202190, and STAT3 inhibitors (**Fig. 6C**). In patients with PAH, MMP-1 expression was significantly increased in hypoxic M1 macrophages compared to that in hypoxic M0 macrophages, whereas the expression of MMP1 in M1 macrophages was significantly suppressed by p42/p44 MAPK, p38 MAPK, JNK-1/2, PI3K/AKT, and STAT3 inhibitors, but not by STAT1 inhibitor (**Fig. 6B**). Hypoxia induced MMP-10 expression in M1 macrophages compared to that in M0 macrophages, while the expression of MMP10 in hypoxic M1 macrophages was significantly suppressed by PD98059, SB202190, SP600125, LY204002, STAT1, and STAT3 inhibitors (**Fig. 6D**). Taken together, p42/p44 MAPK, p38 MAPK, JNK-1/2, PI3K/AKT, and STAT3 are essential for MMP-1 and MMP-10 expression in hypoxia-induced M1 macrophages. These results suggest that the regulation of MMP10, but not MMP-1 expression, may be mediated through a STAT1-dependent pathway in hypoxia-induced M1 macrophages.

## Discussion

This study discovered that MMP-1 and MMP-10 are upregulated in human serum, M1-polarized macrophages, and the lungs obtained from patients with PAH, as well as in experimental PAH lungs. We investigated that the role of MMP-10 in the pathogenesis of PAH using adenovirus-mediated overexpression of active MMP-10. Overexpression of active MMP-10 promotes human PASMC proliferation and migration. Similarly, overexpression of active MMP-10 in PASMCs isolated and cultured from hypoxia and MCT-treated rats also promoted PASMCs proliferation and migration compared to that in the control group. Moreover, we found that PASMCs from MCT- and hypoxia-treated rats were more sensitive to the release of active MMP-10 and vascular remodeling compared to PASMCs from the control group, indicating that MMP-10 plays a vital role in vascular remodeling in clinical and experimental PAH. In an attempt to more closely mimic the patients with PAH linked with a lack of oxygen in the blood, M1-polarized macrophages from patients with PAH were cultured under hypoxic conditions. In PAH patients, M1-polarized macrophages exhibited an increase in MMP-1 and MMP-10 expression in response to hypoxia, which was mediated by distinct MAPK, PI3K/AKT, and STAT3 signaling pathways. However, the STAT1 signaling pathway was observed to regulate MMP-10, but not MMP-1 expression, in hypoxia-induced M1 macrophages. This study provides evidence for the regulatory role of MMP-10 in the pathogenesis of PAH.

MMPs have been identified as key players in vessel remodeling as well as the development and progression of PAH. Wang et al. summarized the specific distribution of MMP-1 in ECs and VSMCs as well as MMP-9 in medial VSMCs and adventitial microvessels 17. However, Lepetit et al. demonstrated that there was no difference in MMP-1 expression in PASMCs between controls and patients with PAH 9. Interestingly, VSMCs have been reported to increase the secretion of MMP-1 when co-cultured with monocyte-derived macrophages (Lee et al., 1995). Macrophages have also been reported to be potential sources of MMPs 2. This study also showed the expression of MMP-1 and MMP-10 increased in human serum, M1-polarized macrophages, and lung specimens from patients with PAH.

Reportedly, MMP-10 is expressed by macrophages in the human lung of patients with cystic fibrosis 18. Circulating MMP-10 is overexpressed in the aortic valve of patients with aortic stenosis 19. In agreement with the findings of previous studies, we found that MMP-1 and MMP-10 levels were increased in the serum of patients with PAH, compared to that in the serum of healthy controls, and highly expressed in the media and adventitia of the human pulmonary artery. By differentiating monocytes from the peripheral blood of patients with PAH into M0, M1, and M2 macrophages, we observed enhanced MMP-9 mRNA expression in monocyte-derived M0, M1, and M2 macrophages from PAH patients and controls. MMP-9 mRNA levels were higher in M0 and M1 macrophages from PAH patients than that in the M0 and M1 macrophages from control groups. These results are consistent with prior observations of elevated MMP-9 serum levels in patients with idiopathic pulmonary fibrosis 20. Furthermore, we found that the mRNA and protein levels of MMP-1 and MMP-10 were higher in M1 macrophages derived from patients with PAH than that in the control groups. Interestingly, there were no significant differences in the MMP-1 and MMP-10 expression on M0 and M2 macrophages derived from the PAH group compared to that in the control, indicating that the secretion of MMP-1 and MMP-10 by macrophages may require an activated state, as exhibited in PAH patients. Consistent with the results a previous study, naïve macrophages did not express MMP-10, but were able to do so upon M0 differentiation and M1 polarization 19.

Given the marked elevation of MMP-10 levels in patients with PAH, it was particularly relevant to study the contribution of MMP-10 to the PASMC phenotype in PAH. In experimental models, we noticed that MMP-10 is upregulated in the lung tissue of MCT- and hypoxia-treated rats, but not in control rats. Moreover, CD68-positive macrophages were MMP-10 positive on double immunofluorescence staining in the lung tissues of MCT- and hypoxia-treated rats, whereas no signal for MMP-10 was detected in the control rat lung tissues, including resident CD68-positive macrophages. These findings demonstrate that MMP-10 is not expressed in the control lung tissues but is induced by macrophages in the lung tissues of MCT- and hypoxia-treated rats, which is consistent with the observed MMP-10 expression on macrophages in the lungs of patients with cystic fibrosis 18. MMP-10 expression in atherosclerotic lesions is restricted to the neointima, mainly in cells with a macrophage-like morphology 21. In contrast, MMP-10 was found to be overexpressed in perivascular macrophages and pulmonary vascular cells from the remodeled walls in patients with systemic sclerosis-associated pulmonary hypertension 22. Although MMP-10 has been reported to be highly expressed in the damaged lung tissue, there are few reports regarding the involvement of MMP-10 in vascular remodeling. Reportedly, PMA-activated macrophages release MMP-1 and MMP-9, which affects the aortic SMC phenotype and neo-angiogenesis 13, suggesting that the overproduction of MMPs by macrophages plays a role in regulating the behavior of VSMCs. MMP-10 inhibition reduced cell proliferation through the downregulation of PCNA and Ki67 mRNA in the lung tissue of Fra-2 transgenic mice, resulting in an intense pulmonary vascular remodeling 22. Our results confirm the previous observation that overexpression of MMP-10 in PASMCs derived from humans as well as MCT- and hypoxia-treated rats promotes cell migration and proliferation through the upregulation of cyclin D1 and PCNA expression. A previous report showed that bradykinin induces cell proliferation via MMP2/9-dependent pro-HB-EGF shedding, which is linked to the upregulation of cyclin D1 in corneal fibroblasts 23. Although it is still not clear whether MMP-10 essentially contributes to the upregulation of proliferation markers or has a more supportive character, the present results suggest that MMP-10 is a possible important contributor to PAH progression and a potential biomarker for PAH.

Although an overexpression of MMP-10 promotes pulmonary vascular remodeling by facilitating cell proliferation and migration in PASMCs, the mechanisms underlying the production of MMP-10 are not fully understood. The MMP-1 and MMP-10 promoters share a certain degree of similarity and harbor several cis-elements, allowing for the regulation of MMP gene expression by a diverse set of trans-activators, which includes AP-1 and STAT binding sites 24. Several reports have shown that AP-1 and STAT transcription factors appear to be regulated by multiple signal transduction pathways, including MAPKs (ERK, JNK, and p38 MAPK), PI3K/AKT, STAT1, and STAT3 signaling pathways 15, 25, 26. Our study shows that during the response to hypoxia, p42/p44 MAPK, p38 MAPK, JNK1/2, PI3K/AKT, and STAT3 are involved in the regulation of MMP-1 and MMP-10 expression in monocyte-derived M1 macrophages from patients with PAH. We also noticed that the specific regulation of MMP-10, but not MMP-1, is mediated by a STAT1-dependent pathway. STAT3 exerts pleiotropic effects on the biology of interaction and diseases pathogenesis, as compared to STAT1 27. Although inhibiting STAT3 effectively decreases the expression of MMP-1 and MMP-10 in monocyte-derived M1 macrophages, we believe that STAT3 blockage also inhibits biological processes. Brian Saunders et al. have shown that MMP-1 activation is mediated by MMP-10 and that it induces human capillary tubular network collapse 28, suggesting that MMP-10 functions as a key protease in the regulation of vascular cell behaviors by modulating MMP-1 activation. The selective inhibitor of STAT1 inhibits MMP-10 expression in monocyte-derived M1 macrophages and may cause MMP-1 inactivation, suggesting that STAT1 may be a therapeutic target for MMP regulation and vascular remodeling.

In summary, this study demonstrates that MMP-10 and MMP-1 levels are overexpressed in monocyte-derived M1 macrophages from patients with PAH. In the experimental PAH models, we noticed that MMP-1 and MMP-10 were mostly expressed in CD68-positive cells in the lung tissue. Mechanistically, we demonstrated that the expression of STAT1-dependent MMP-10 controls PASMC phenotypes that are characterized by increased cell proliferation and migration. In conclusion, our study presents a potential therapeutic strategy for PAH by using STAT1 as a target to regulate MMP-10 levels.

## Materials and Methods

### Materials

PD98059, SB203580, SP600125, and LY294002 were obtained from Biomol (Plymouth Meeting, PA, USA). Fludarabine (STAT1 inhibitor) was from Selleckchem (Houston, TX, USA). S3I-201 (STAT3 inhibitor) was from Abcam (ab141434, Cambridge, MA, USA).

### Human Subjects

Medical records of 39 patients (9 males, 30 females; mean age 56.3±13.7 years) with PAH were collected in the Department of Critical Care Medicine, Kaohsiung Veterans General Hospital, while 30 healthy volunteers (7 males, 23 females; mean age 54.0±18.2 years) were recruited as controls. The PAH group consisted of patients with pulmonary arterial systolic pressure (PASP) of ≥20 mmHg by right heart catheterization and normal pulmonary capillary wedge pressure ≤15 mmHg 29. All work involving human samples from patients with PAH or healthy volunteers was approved by the Human Research Committee of Kaohsiung Veterans General Hospital. Informed written consent was obtained from all participants at the time of the sample collection.

### Isolation, invite differentiation, and stimulation of monocytes and monocyte-derived macrophages

Monocytes were isolated from peripheral blood donated from healthy individuals or patients with PAH. Purified monocytes were cultured in Macrophage Culture Medium (MCM) plus 20 ng/mL of macrophage colony-stimulating factor (M-CSF) (#216-MC, R & D system, Minneapolis, MN, USA) for 7 days to differentiate into M0 macrophages. For M1 and M2 polarization experiments, at 7^th^ day, the MCM was removed and replaced with MCM supplemented with 5% (v/v) fetal bovine serum (FBS; Thermo Fisher, Waltham, MA, USA). The recombinant human INF-γ 10 ng/mL (#285-IF, R&D Systems, Minneapolis, MN, USA) and LPS 200 ng/mL (#L2654, Sigma-Aldrich, St. Louis, MO, USA) were added in the medium for additional 18 hours to induce classical activated macrophages (M1), medium with recombinant human interleukin-4 (IL-4) 20 ng/mL (#204-IL, R&D system, Minneapolis, MN, USA) to induce alternative activated macrophages M2 30.

### PAH models

A hypoxia-induced and a MCT-induced rat PAH model were used in our study and the experimental protocols were approved by the Institutional Animal Care and Use Committee, Kaohsiung Veterans General Hospital. Male Sprague-Dawley (SD) rats were purchased from BioLASCO (Ilan, Taiwan) and were handled according to the IACUC guidelines. week). There were 10-12 rats in each experimental group. For the hypoxia-induced rat PAH model, 200-230g, 6-week-old SD rats were exposed to room air (normoxia) or 10% oxygen (hypoxia) in an anaerobic chamber (Coy Laboratory Products, MI, USA) for 8 weeks, and the oxygen concentration was monitored by an oxygen controller (Coy Laboratory Products, MI, USA). For MCT-induced PAH studies, SD rats were randomly given a subcutaneous injection of either 60 mg/kg MCT (Sigma-Aldrich, St. Louis, MO, USA) or 0.9% saline (vehicle). Animals were sacrificed at day 28, and PAH pathology was assessed as described previously 31. After the treatment, RVSP was measured by right heart catheterization using a polyethylene-50 tubing connected to a 24-gauge needle through the PowerLab/8SP (AD Instruments Ltd. Dunedin, New Zealand). A weight ratio of the RV divided by the sum of LV and S (RV/[LV + S]) was determined as an index for right ventricular hypertrophy.

### Isolation and culture of human and rat PASMC

Human PASMCs were purchased from ATCC (PSC-100-023, Manassas, VA, USA). For PASMCs isolated from MCT- and hypoxia-induced rats, the branches of the main pulmonary artery were isolated from the rat lung and cleared of connective tissue. The endothelium was removed by gently rolling the luminal surface with a cotton swab. PASMCs were then enzymatically isolated and cultured in medium 231 (M231, Thermo Fisher, Waltham, MA, USA) containing smooth muscle growth supplement (SMGS, Thermo Fisher, Waltham, MA, USA)^32^. The purity of the PASMCs in the primary cultures was confirmed via immunofluorescence staining using a specific antibody against smooth muscle α-actin. Cells at 80% confluence were used for all experiments. Experiments were performed using cells from passages 3 to 8.

### Histology and Immunohistochemical Analysis of Pulmonary Arteries

Each lung tissue sample was fixed in 4% formalin and embedded in paraffin blocks. Tissue sections (4 μm) were subjected to Elastica van Gieson (EVG) staining or immunohistochemistry using anti-MMP-1(1:100, 10371-2-AP, Proteintech Group, Chicago, IL) and anti-MMP-10 (1:100, MAB9101, R&D Systems, Minneapolis, MN, USA) antibody, followed by anti-rabbit HRP secondary antibody (Santa Cruz Biotechnology, Santa Cruz, CA). For immunofluorescence, lung sections were stained using rabbit anti-MMP-1, mouse anti-MMP-10, or rabbit anti-CD68 (1:100, 25747-1-AP, Proteintech Group, Chicago, IL), and mouse anti-CD68 (1:100, ab31630, Abcam, Cambridge, UK) for 1 h, followed by incubation with Alexa 488-labeled anti-rabbit, Alexa 488-labeled anti-mouse, Alexa 568-labeled anti-rabbit, or Alexa 568-labeled anti-mouse (Thermo Fisher, Waltham, MA, USA) for 1 h. Lung sections were mounted with the antifade reagent in the presence of 4′,6-diamidino-2-phenylindole (DAPI) (Invitrogen, Waltham, MA, USA). Images were acquired using LEICA microscopy (Leica, Wetzlar, Germany) and quantified with Image J analysis software (NIH, Bethesda, MD, USA).

### Semi-quantitative polymerase chain reaction

Total RNA from cells was extracted using TRIzol (Thermo Fisher, Waltham, MA, USA). Two micrograms of purified RNA were reverse transcribed to cDNA using a High-Capacity cDNA Reverse Transcription kit (Applied Biosystems, Foster City, CA, USA). Real-time polymerase chain reaction (PCR) was performed with Fast SYBR Green Master Mix (Applied Biosystems, Foster City, CA, USA) on a StepOnePlus System (Applied Biosystems, Foster City, CA, USA). The following primer sets were used: human MMP-1 forward, 5'-ATG AAG CAG CCC AGA TGT GGA G-3' and reverse, 5'-TGG TCC ACA TCT GCT CTT GGC A-3'; MMP-3 forward, 5'-CAC TCA CAG ACC TGA CTC GGT T-3' and reverse, 5'-AAG CAG GAT CAC AGT TGG CTG G-3'; MMP-7 forward, 5'-TCG GAG GAG ATG CTC ACT TCG A-3' and reverse, 5'-GGA TCA GAG GAA TGT CCC ATA CC-3'; MMP-8 forward, 5'-CAA CCT ACT GGA CCA AGC ACA C-3' and reverse, 5'-TGT AGC TGA GGA TGC CTT CTC C-3'; MMP-9 forward, 5'-GCC ACT ACT GTG CCT TTG AGT C-3' and reverse, 5'-CCC TCA GAG AAT CGC CAG TAC T-3'; MMP-10 forward, 5'-TCC AGG CTG TAT GAA GGA GAG G-3' and reverse, 5'-GGT AGG CAT GAG CCA AAC TGT G-3'; MMP-11 forward, 5'-TCC AGG CTG TAT GAA GGA GAG G-3' and reverse, 5'-GGT AGG CAT GAG CCA AAC TGT G-3'; MMP-12 forward, 5'-GAT GCT GTC ACT ACC GTG GGA A-3' and reverse, 5'-CAA TGC CAG ATG GCA AGG TTG G-3'; MMP-14 forward, 5'-CCT TGG ACT GTC AGG AAT GAG G-3' and reverse, 5'-TTC TCC GTG TCC ATC CAC TGG T-3'; MMP-25 forward, 5'-TGA CAA GCC CAC AAG GAA ACC C-3' and reverse, 5'-GAT GGC GTC AAA ATT GCC CTC AC-3'; COX-2 forward, 5'-CGG TGA AAC TCT GGC TAG ACA G-3' and reverse, 5'-GCA AAC CGT AGA TGC TCA GGG A-3'; CD206 forward, 5'- AGC CAA CAC CAG CTC CTC AAG A-3' and reverse, 5'- CAA AAC GCT CGC GCA TTG TCC A-3'; GAPDH forward, 5'- GTC TCC TCT GAC TTC AAC AGC G-3' and reverse, 5'- ACC ACC CTG TTG CTG TAG CCA A-3'. Values were normalized to GAPDH mRNA levels and are presented as 2^-∆CT^.

### Western blot

Proteins were resolved on 10% SDS-PAGE gels, electroblotted onto a PVDF membrane, and probed using primary antibodies against MMP-1 (1:1000), MMP-10 (1:1000), cyclin D1 (1:1000, #2978, Cell Signaling Technology, Danvers, MA, USA), PCNA (1:1000, ab2426, Abcam, Cambridge, MA, USA), and β-actin (1:2000, ab8227, Abcam, Cambridge, MA, USA). After 16 h, the membranes were incubated with HRP-conjugated second antibody, and chemiluminescence (ECL, Cell Signaling Technology, Danvers, MA, USA) was employed to visualize the protein bands. Band intensities were analyzed densitometrically using Image J analysis software (NIH, Bethesda, MD, USA).

### Enzyme-linked immunosorbent assay (ELISA)

The MMP-1 and MMP-10 levels in the serum were measured by MMP-1 and MMP-10 ELISA Ready-SET-Go^®^ kit (eBioscience, San Diego, CA, USA) according to the manufacturer's protocol.

### Adenovirus-mediated MMP-10 gene transfer in human and rat PASMC

A recombinant adenovirus containing human MMP-10 gene (Adv-MMP-10) was kindly provided by Dr. Graciela Sala-Newby (Bristol Heart Institute, University of Bristol, Bristol, UK). Cells were infected with Adv-MMP-10 (1, 10, 20 multiplicity of infection, MOI) for 48 or 72 h in complete cell culture medium. Subsequently, cells were washed with PBS and then cultured in supplement free culture medium for additional 48 h, and then cells and culture medium were collected and overexpression efficiency was confirmed via Western blotting.

### Cell viability and proliferation

Cell viability was determined by Alamar blue assay. Briefly, cells were plated into a 96-well plate at the concentration of 3 × 10^4^/mL. At different time point after Adv-MMP-10 infection, AlamarBlue reagent was introduced to each well at a volume of 10% of total well volume, and allowed to incubate for 2 h. Absorbance readings at wavelengths of 570 nm and 600 nm were compared to the absorbance of negative control wells containing media and AlamarBlue only.

Cell proliferation was determined using a BrdU labeling kit (Roche, Indianapolis, IN, USA). The BrdU incorporation was detected by FITC-BrdU antibody and analyzed under a fluorescent microscope. The experiments were performed in triplicate.

### Wound healing assay

Cells were seeded on 6 well plates and cells were allowed to grow to complete confluence. After Adv-MMP-10 infection for 48 h, cells was washed with PBS and then treated with 10μg/ml mitomycin C for 2 h prior to wounding. Immediately following scratch wounding (0 h) and after incubation of cells for 24, 48, or 72 h, phase-contrast images of the wound healing process were photographed digitally with an inverted microscope. The distance of the wound areas were measured on the images, set at 100% for 0 h, and the mean percentage of the total distances of the wound areas was calculated using Image J analysis software (NIH, Bethesda, MD, USA).

### Statistical analysis

Statistical analysis of experimental data was carried out using GraphPad Prism 7 (GraphPad Software, La Jolla, CA). Parametric analysis of normally distributed data was performed by ordinary one-way analysis of variance (ANOVA) using Dunnett's multiple comparisons test. Nonparametric data were analyzed using the Kruskal-Wallis test with Dunn's multiple comparisons test. Multiple-group analysis was performed by ordinary two-way ANOVA using the Holm-Sidak multiple comparisons test. Results are expressed as mean ± SD from at least three experiments. Significant differences are indicated by * (P < 0.05), and very significant differences are indicated by # (P < 0.01).

## Figures and Tables

**Table 1:** The clinical characteristics of PAH patients and controls (healthy donors).

	Control (n=39)	PAH (n=30)	P-value
Age, years	56.3±13.7	54.0±18.2	0.552
Gender, female	30 (76.9%)	23 (76.6%)	0.980
mPAP, mmHg	-	42.5±15.7	-
PVR, Wood units	-	6.5±5.4	-
Cardiac index, L/min/m2	-	3.5±1.6	-
Cardiac output, L/min	-	5.1±2.1	-
VO2 peak	-	48.9±12.7	-
6MWD , m	-	288.4±106.4	-

Data were showed as mean±SD or the number (%). mPAP indicates mean pulmonary artery pressure; PVR, pulmonary vascular resistance; 6MWD, six-minute walk distance.
